# The clinical effect of manual therapy including Tuina (or Chuna) on post-stroke shoulder hand syndrome–a literature review

**DOI:** 10.3389/fneur.2026.1820005

**Published:** 2026-05-12

**Authors:** Ga-Young Kim, Won-Suk Sung, Eun-Jung Kim

**Affiliations:** 1 Department of Korean Medicine, Graduate School, Dongguk University, Seoul, Republic of Korea; 2 Department of Acupuncture & Moxibustion, Dongguk University Bundang Oriental Hospital, Seongnam-si, Gyeonggi-do, Republic of Korea

**Keywords:** Chuna manual therapy, literature review, manual therapy, post-stroke, shoulder-hand syndrome

## Abstract

**Background:**

Post-stroke SHS is a common sequela of stroke, presenting with pain, hypersensitivity, allodynia, and edema in the upper extremities. These symptoms can significantly hinder functional recovery and reduce quality of life. Manual therapy, including Tuina (or Chuna) has been increasingly applied in integrative rehabilitation; however, its clinical role has not been systematically evaluated.

**Methods:**

We conducted a comprehensive search across 13 databases, including PubMed, Cochrane Library, Embase, Wanfang Data, China National Knowledge Infrastructure (CNKI), KoreaMed, Korean Medical Database (KMBase), Korean Studies Information Service System (KISS), ScienceON, KoreaScience, DBPia, Research Information Sharing Service (RISS), and Oriental Medicine Advanced Searching Integrated System (OASIS). Studies were selected according to predefined criteria. We extracted and analyzed the characteristics of the selected studies to evaluate their clinical efficacy and safety.

**Results:**

All 13 selected studies were randomized controlled trials (RCTs) that applied Tuina manual therapy. These interventions were additionally interpreted within the framework of Chuna manual therapy. Among these, 1 study employed lymphatic drainage massage as the manual therapy, while the remaining 12 studies targeted specific body areas or acupuncture points. Based on the treatment approaches, we classified the studies into 4 categories: monotherapy (1 study), add-on therapy with traditional Chinese medicine (2 studies), add-on therapy with conventional treatment (7 studies) or combination therapy (3 studies). All studies reported significant effects across various outcome measures, while only 2 evaluated the occurrence of adverse events.

**Conclusion:**

This research suggests the potential clinical value of manual therapy including Tuina (or Chuna) into SHS. However, there was variability in the duration of therapies, techniques, acupuncture points, and outcome measures, which made it difficult to generalize the results. To develop standardized guidelines for manual therapy in SHS, further large-scale, multinational studies are needed.

## 1 Introduction

Shoulder-hand syndrome (SHS) is a sequela of stroke characterized by severe pain, edema, alternations in skin temperature and color, and motor dysfunction of the upper extremity. SHS typically occurs within 1 to 6 months following a stroke and significantly impacts both the quality of life and functional recovery by delaying rehabilitation (1, 2). SHS is classified within the spectrum of Complex Regional Pain Syndrome (CRPS) (3) and is specifically categorized as CRPS type I, also known as reflex sympathetic dystrophy, due to the absence of identifiable nerve damage (4).

The prevalence of SHS ranges from 2% to 49% (5), with a reported domestic prevalence of 15.6% as of 2024 (6). The pathophysiology of SHS remains incompletely understood, but proposed contributing factors include scapulohumeral instability, overactivity of the sympathetic nervous system, alterations in peripheral nerves, and localized inflammation (7). There are currently no established treatment guidelines for SHS. Management strategies aim primarily at pain alleviation and preservation of joint mobility (1). These strategies encompass pharmacological interventions such as anti-inflammatories, analgesics, antidepressants, muscle relaxants, and anticonvulsants, as well as sympathetic nerve blocks and various therapeutic modalities including passive exercises and contrast baths (8).

In East Asian countries, manual therapy has developed under different systems, such as Tuina in China and Chuna in Korea. Although Chuna shares historical backgrounds with Tuina, it has evolved into a distinct system with its own classification framework and clinical applications. Chuna therapy involves manual manipulation by a practitioner to stimulate and promote the regeneration of damaged tissues and restore previous function (9). Initially targeted at musculoskeletal disorders (10), Chuna therapy has recently expanded to address conditions such as Tourette syndrome (11), Pediatric fever (12), Postpartum lactation insufficiency (13), and Rhinitis (14).

The 2021 Standard Clinical Guidelines for Stroke in Korean Medicine (15) provided evidence for Korean medical interventions based on clinical symptoms of stroke, though recommendations were limited to moxibustion for SHS. In the study by Oh & Lee (16), a systematic review demonstrated that Chuna therapy has a significant effect on shoulder pain in stroke patients with hemiparesis. Given that SHS is identified as one of the major factors contributing to shoulder pain in hemiparetic patients (17), this finding suggests the potential for the proactive use of Chuna therapy in managing SHS. Lin et al. (18) conducted a systematic review in 2017 on the effects of massage (推拿) for SHS, although most studies combined this with acupuncture and other traditional medical treatments, presenting a limitation.

This study aims to analyze and review domestic and international research on the application of manual therapy for post-stroke SHS to assess the clinical status, effectiveness, and safety of such treatments.

## 2 Methods

### 2.1 Ethics

Due to the absence of personal information collection from patients, ethical approval was not necessary.

### 2.2 Search strategy

A comprehensive literature search was conducted using both domestic databases (KoreaMed, Korean Medical Database, Korean Studies Information Service System, ScienceON, KoreaScience, DBPia, Research Information Sharing Service, and Oriental Medicine Advanced Searching Integrated) and international databases (PubMed, Cochrane Library, Embase, China National Knowledge Infrastructure, and Wanfang Data). The search strategy combined controlled vocabulary terms (including Medical Subject Headings, where applicable) and free-text terms for both the condition and the intervention. For the condition, the following terms were used: “reflex sympathetic dystrophy,” “complex regional pain syndrome type I,” “sudeck atrophy,” “algodystrophy,” “shoulder hand syndrome,” and “shoulder–hand syndrome.” For the intervention, database-specific terms were applied: “Tuina” was used for Chinese databases, while “Chuna” was used for Korean databases, along with related terms such as “massage,” “manipulation,” and “manual therapy.” Equivalent Chinese and Korean terms were additionally used for regional databases. No restrictions were placed on publication year or language.

Searches were performed up to February 2, 2026, from the initiation date of each database, and relevant literature was reviewed and further confirmed.

### 2.3 Inclusion and exclusion criteria

The literature was selected based on the following inclusion and exclusion criteria.

#### 2.3.1 Study design

Only randomized controlled trials (RCTs) were included. Studies not classified as clinical research, such as experimental or review articles, were excluded.

#### 2.3.2 Subjects

Studies involving patients with post-stroke SHS were selected.

#### 2.3.3 Treatment

Manual therapies performed by practitioners using their hands, including Chuna, Tuina, acupressure, and massage, were included. Studies were included only if the applied manual techniques and underlying theoretical principles were consistent with those of Tuina or Chuna. Studies in which manual therapy involved instrument-assisted techniques were excluded. In addition, studies combining manual therapy with other traditional medical treatments, such as acupuncture and moxibustion, which have been reported to demonstrate therapeutic effects when used in combination (15, 16) were also excluded to isolate the specific effects of manual therapy.

Chuna and Tuina include the 24 traditional techniques such as one-finger scrubbing/pushing thumb (YiZhiChanTui), circular kneading (Mo), rubbing/linear-rubbing (Ca), palm twisting/rubbing with two palms (Cuo), slipping/wipping (Mo), pinching (Nie), pinching spine (NieJi), patting (Pai), tapping/knocking (Ji), flicking/shaking (Dan), pressing (An), grasping (Na), pushing (Tui), vibrating (Zhen), rolling (Gun), kneading (Rou), finger-nail pressing (Qia), shaking (Dou), rotating (YaoDong), finger twisting/holding (NianJi), traction (QianYin), manipulation (Ban), foot treading (CaiQiao), and piggyback ride traction (Bei). Papers utilizing traditional techniques were not excluded (9).

### 2.4 Study selection and data extraction

One researcher (KGY) conducted the initial screening based on the title and abstract according to predefined criteria. Discrepancies were resolved through discussion with a second researcher (SWS) to reach a final decision. Subsequently, full texts were obtained and studies meeting the criteria were selected. Data extraction included basic information (study type, first author, publication year, and country), specific features of the study (study design, intervention methods for experimental and control groups), assessment scales, and outcomes. In cases where sufficient information was not available, analyses were performed with the available data.

## 3 Results

### 3.1 Study selection

**Figure 1:**
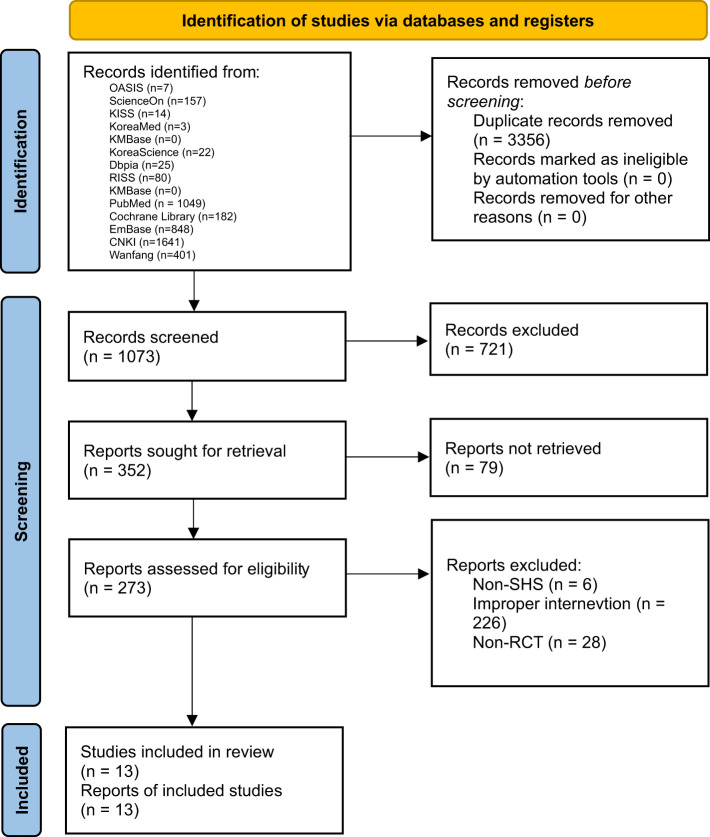
Flowchart of search and selection process of research.

As a result of the database search, a total of 4,429 papers were identified. After duplicate removal, a total of 1,073 records were screened by title and abstract, of which 721 were excluded. The full texts of 352 reports were sought for retrieval, and 79 could not be obtained. Consequently, 273 reports were assessed for eligibility. Of these, 260 were excluded for the following reasons: studies that did not involve patients with SHS (*n* = 6), ineligible interventions (*n* = 226), and non-randomized study designs (*n* = 28). Ultimately, 13 studies met the inclusion criteria and were included in the final review (Figure 1).

### 3.2 Characteristics of the included studies

All 13 selected studies were RCTs, published between 2002 and 2023, and all were published in China. Among the 13 included studies, 1 used manual therapy as a sole intervention in the experimental group (19), whereas the remaining 12 applied manual therapy in combination with other treatments. Of these 12 studies, 2 adopted an add-on design in which traditional Chinese medicine interventions such as acupuncture and electroacupuncture were administered to the control group and manual therapy was additionally provided to the experimental group (20, 21). Seven studies added manual therapy to conventional interventions, including hyperbaric oxygen therapy, taping, and rehabilitation therapy (22–28). In three studies, both the experimental and control groups received one of the conventional post-stroke treatments as co-interventions; the experimental group additionally underwent manual therapy alone, whereas the control group received other therapies instead of manual therapy (29–31).

#### 3.2.1 Subject selection

The 13 selected studies focused on stroke patients with SHS. A total of 1,091 SHS patients were included in the studies, with 576 allocated to the experimental group and 515 to the control group through random assignment. Among the 1,091 participants, 586 were male and 505 were female.

#### 3.2.2 Control group settings

In 8 studies (22–27, 29, 31), conventional post-stroke care was provided to both the experimental and control groups as basic treatment. The detailed intervention was not described in one study (31). Medication was the most frequently used co-intervention, reported in 6 studies (22–26, 29). The stated purposes of pharmacological treatment included neurological management, symptomatic treatment and prevention of complications, as well as improving microcirculation, providing nutritional support, protecting brain cells, and maintaining electrolyte balance. Three studies administered physical therapy or occupational therapy (25, 27, 29) and 1 study administered conventional nursing care (24).

Five studies (26–30) used conventional interventions consisting of rehabilitation training for the control group. The training protocols were described in all studies except one (28), although the specific methods varied across studies. Rehabilitation training included, for example, joint exercises, contrast baths (warm and cold water), and postural training.

Four study applied conventional treatments such as ultrasonic therapy with intramuscular effect sticking (22), hyperbaric oxygen therapy (23), physical therapy (24) and suspension movement (25). In addition, 2 study provided non-manipulative interventions consisting of irregular passive or voluntary movements to the control group (19, 31).

Two studies only utilized traditional Chinese medicine treatments as control interventions, specifically employing electroacupuncture (20) and acupuncture (21). In 2 studies, the control group was treated with rehabilitation training in combination with traditional Chinese medical interventions, such as electroacupuncture and herbal fumigation (29, 30).

#### 3.2.3 Experimental group interventions

All studies detailed the intervention methods for the experimental groups. One study (20) specifically utilized lymphatic drainage manipulation as the manual therapy, while the remaining 12 studies applied manual therapy to specific areas or acupoints of the body. One study limited the treatment area to the cervical region (27), whereas the other 12 studies targeted the entire upper extremity.

Chuna manual therapy was applied as part of the manual therapy intervention in all included studies. Traditional Chuna manual therapy (TCMT) was applied in 12 studies (19–23, 25–31), and comprised 13 techniques, including grasping, pinching, pressing, kneading, circular kneading, rolling, rotating, palm twisting/rubbing with two palms, finger twisting/holding, shaking, slipping/wipping, pushing, pinching, and patting. In addition, fascia Chuna manual therapy (FCMT) was used in 3 studies (24, 27, 28), joint-mobilizing Chuna manual therapy (JCMT) in 8 studies (19, 24–26, 28–31), and dislocation Chuna manual therapy (DCMT) in 1 study (31). Among the studies employing acupressure, 8 studies (21–23, 25, 26, 28–30) used acupoints located on the spine and upper limb.

#### 3.2.4 Evaluation criteria

Upper limb motor function was most commonly assessed using the Fugl–Meyer Score (FMS), which was applied in 9 studies (20, 21, 23–26, 28–30). For pain evaluation, the visual analog scale (VAS) was used in 9 studies (20–26, 28, 30), the numeric rating scale (NRS) in 1 study (29), and the Canadian McGill Pain Questionnaire (MPQ) in 1 study (27). Overall SHS-related symptoms were evaluated using the Shoulder–Hand Syndrome Score (SHSS) in 2 studies (22, 29). Range of motion outcomes included shoulder adduction angle (SAA) (21) and total passive range of motion (TPRM) (20). Muscle spasticity of the upper limb was assessed using the Ashworth Scale (AS) in 1 study (21). Edema was evaluated either by the drainage method (20, 23, 27) or by edema grading scales (28). The Barthel Index (BI) for activities of daily living was used in 2 studies (20, 25). In the study that applied hyperbaric oxygen therapy, microcirculation at the ring fingernail was measured (23). The total effective rate was reported in 7 studies (19–22, 24, 29, 31).

#### 3.2.5 Treatment effects

Due to the clinical and methodological heterogeneity among the included studies, a narrative synthesis was conducted. The included studies were categorized into four groups according to the type of intervention: monotherapy, traditional Chinese medicine add-on therapy, conventional treatment add-on therapy, and combination therapy. Data were extracted and summarized as reported in the original articles.

(1) Monotherapy (1 Study)

Dong et al. (19) included 308 patients who met the diagnostic criteria for SHS. During the 3-month treatment period, the experimental group received TCMT and JCMT, whereas the control group underwent non-manipulative therapy consisting of irregular passive or voluntary movements. ER was significantly higher in the experimental group than in the control group (*p* < 0.001) (Table 1).

**Table 1:** The analysis of 1 included study that conducted manual therapy including Chuna as Monotherapy.

Author (year)	Number of SHS patients (Male/Female)		Age		Duration	Intervention	Control	Outcome measurements	Results		
									EG	CG	*P* value
Dong et al. (2002) (19)	EG	CG	EG	CG	3 months	BID, 30 min 1) TCMT - pressing and circular kneading 2) JCMT - passive movements of upper limb	Non-manipulation - Irregular passive movement or auronomic movement	ER	32.8%	4.6%	p < 0.001
	180 (106/74)	128 (77/51)	NR	NR				AE	NR	NR	

AE, Adverse Event; BID, Bis In Die; CG, Control Group; DCMT, Dislocation Chuna Manual Therapy; EG, Experimental Group; ER, Effective rate; NR, No Result; TCMT, Traditional Chuna Manual Therapy; JCMT, Joint-mobilizing Chuna manual therapy.

(2) Add-on therapy with Traditional Chinese medicine (2 Studies)

Hong (20) randomized 60 patients aged 36–69 years using a random number table and compared TCMT-based lymphatic drainage manipulation with electroacupuncture over 4 months. The experimental group showed significantly greater improvements in pain, upper limb motor function, activities of daily living, range of motion, and edema than the control group (*p* < 0.05).

Yao (21) included 98 post-stroke patients with SHS aged 43–79 years and compared TCMT with acupuncture for 1 month. Significant between-group differences were observed in pain reduction, motor function, shoulder range of motion, and spasticity in favor of the experimental group (*p* < 0.05).

Overall, both studies in this category reported significantly greater improvements in the experimental groups than in the control groups. When reported, the total effective rate was consistently higher in the experimental groups (Table 2).

**Table 2:** The analysis of 2 included studies that conducted manual therapy including Chuna as Add-on therapy with traditional Chinese medicine.

Author (year)	Number of SHS patients (Male/Female)		Age		Duration	Intervention	Control	Outcome measurements	Results		
									EG	CG	*P* value
Hong (2021) (20)	EG	CG	EG	CG	4 months (5 days a week)	QD, 30 min Control+ 1) Lymphatic drainage manipulation (TCMT - pressing and circular kneading)	Electroacupuncture	ER	29%	24%	*p* < 0.05
	30 (12/18)	30 (14/16)	50.15 ± 4.17	49.42 ± 4.96				VAS	6.91 ± 0.13 → 2.31 ± 0.17	6.92 ± 0.16 → 3.39 ± 0.21	*p* < 0.05
								Edema (mL)	20.13 ± 2.61 → 12.13 ± 2.79	20.17 ± 2.29 → 16.37 ± 2.47	*p* < 0.05
								FMS	21.13 ± 2.63 → 30.13 ± 3.69	21.17 ± 2.11 → 26.64 ± 2.79	*p* < 0.05
								BI	31.13 ± 3.61 → 55.97 ± 5.19	31.57 ± 3.61 → 46.13 ± 4.93	*p* < 0.05
								TPRM (°)	183.13 ± 18.61 → 193.61 ± 19.47	182.97 ± 18.63 → 227.46 ± 22.97	*p* < 0.05
								AE	NR	NR	
Yao (2019) (21)	EG	CG	EG	CG	1 month	BID, less than 30 min Control+ 1) TCMT - pressing acupoint for 3–5 min (TE14, LI15, HT1, LI11, SI11, 肩前, SI3, EX-UE9, SI9, TE5, and LI4	Acupuncture	FMS	12.13 ± 2.72 → 32.15 ± 4.71	12.96 ± 2.16 → 25.41 ± 2.29	*p* < 0.001
	49 (27/22)	49 (26/23)	43–79	43–78				SAA (°)	25.12 ± 0.72 → 35.55 ± 2.26	25.17 ± 0.71 → 30.03 ± 0.52	*p* < 0.05
								VAS	6.37 ± 1.71 → 1.18 ± 0.21	6.34 ± 1.12 → 2.41 ± 1.09	*p* < 0.05
								AS	2.97 ± 0.21 → 1.02 ± 0.11	2.81 ± 0.24 → 1.42 ± 0.21	*p* < 0.05
								ER	46%	36%	*p* = 0.006
								AE	NR	NR	

AE, Adverse Event; AS, Ashworth Scale; BI, Barthel Index; BID, Bis In Die; CG, Control Group; EG, Experimental Group; ER, Effective rate; FMA, Fugl-Meyer Score; NR, No Result; QD, Quaque Die; SAA; Shoulder Abduction Angle; TCMT, Traditional Chuna Manual Therapy; TPM, Total Passive Range of Motion; VAS, Visual Analog Scale.

(3) Add-on therapy with Conventional treatment (7 Studies)

Yu (22) randomized 72 patients using a random number table and provided medication to all participants for 20 days. The control group received ultrasonic therapy combined with intramuscular effect sticking, whereas the experimental group additionally received TCMT. The add-on TCMT resulted in significantly greater reductions in pain and SHS severity compared with the control treatment alone (*p* < 0.05).

Liu (23) provided medication as basic treatment for 25 days to all participants. The control group received hyperbaric oxygen therapy, whereas the experimental group received additional TCMT. The experimental group demonstrated significantly greater improvements in pain, upper limb motor function, edema, and microcirculation (*p* < 0.05).

Li & Chen (24) provided conventional nursing care and medication for four treatment cycles to both groups. The control group received physical therapy, whereas the experimental group received additional JCMT and FCMT. Significantly greater improvements in pain and motor function were observed in the experimental group (*p* < 0.05).

Cheng (25) administered conventional treatment (medication, physical therapy, and occupational therapy) to all participants aged 44–73 for 28 days. The control group received suspension movement training, whereas the experimental group received additional TCMT and JCMT. The experimental group showed significantly greater improvements in pain, motor function, and activities of daily living (*p* < 0.05).

Lu (26) randomized 64 patients aged 40–66 using a random number table and provided medication to all participants for 29 days. The control group received rehabilitation training, whereas the experimental group received additional TCMT and JCMT. The experimental group demonstrated significantly greater reductions in pain and improvements in motor function (*p* < 0.05).

Sun (27) randomized 34 patients aged 30–60 years using a random number table and provided physical therapy to all participants. The control group received rehabilitation training, whereas the experimental group received additional TCMT and FCMT combined with cervical exercise. Significant reductions in pain and edema were observed in the experimental group compared with the control group (*p* < 0.05).

Liu et al. (28) provided routine rehabilitation training for three treatment cycles to both groups. The experimental group received additional TCMT, JCMT, and FCMT. The experimental group showed significantly greater improvements in pain, motor function, and edema than the control group (*p* < 0.05).

Overall, all studies in this category reported significantly greater improvements in the experimental groups than in the control groups. When reported, the total effective rate was consistently higher in the experimental groups (Table 3).

**Table 3:** The analysis of 7 included studies that conducted manual therapy including Chuna as Add-on therapy with conventional treatments.

Author (year)	Number of SHS patients (Male/Female)		Age		Duration	Intervention	Control	Outcome measurements	Results		
									EG	CG	P value
Yu (2023) (22)	EG	CG	EG	CG	20 days	^*^Medication EOD, 15 min Control+ TCMT - rolling, pushing, pressing, palm twisting/rubbing with two palms, and slipping/wipping - acupoints: three yang meridians of the hand, three yin meridians of the hand, ashi points, LI15, LI11, LI10, TE5, and LI4	^*^Medication Ultrasonic therapy Intramuscular effect sticking	VAS	7.47 (p2 5= 6.25, p75 = 8.00) → 0.89 (p25 = 0.00, p75 = 2.00)	7.33 (p25 = 6.25, p75 = 8.00) → 5.00 (p25 = 4.00, p75 = 6.00)	*p* < 0.01
	36 (21/15)	36 (14/22)	61.83 ± 8.28	62.81 ± 6.41				SHSS	10.14 (p25 = 9.00, p75 = 11.00) → 4.94 (p25 = 4.00, p75 = 6.00)	9.94 (p25 = 9.00, p75 = 11.00) → 6.56 (p25 = 6.00, p75 = 7.0)	*p* < 0.01
								ER	91.7%	86.1%	*p* = 0.038
								AE	None	None	
Liu (2018) (23)	EG	CG	EG	CG	25 days 2 cycles (10 sessions/ cycle, 5-day rest)	^*^Medication QD Control+ TCMT - rolling, pressing, kneading, circular kneading, rotating, and palm twisting/rubbing with two palms - acupoints: LI15, LI14, LU5, LI11, LI10, and LI4	^*^Medication Hyperbaric oxygen	VAS	6.68 ± 2.13 → 2.56 ± 1.45	6.56 ± 2.21 → 4.27 ± 1.52	*p* < 0.05
	30 (17/13)	30 (11/19)	61.8 ± 8.21	62.7 ± 8.61				FMS	28.7 ± 2.3 → 51.4 ± 6.5	29.2 ± 2.6 → 38.2 ± 5.6	*p* < 0.05
								Edema (mm^3^)	24.8 ± 2.6 → 5.2 ± 1.2	25.1 ± 2.8 → 11.6 ± 3.4	*p* < 0.01
								NMS	3.32 ± 0.78 → 1.89 ± 0.44	3.34 ± 0.71 → 2.28 ± 0.52	*p* < 0.05
								AE	NR	NR	
Li and Chen (2018) (24)	EG	CG	EG	CG	4 cycles (10 days/cycle)	^*^Conventional nursing care ^*^Medication QD, 20–30 min Control+ JCMT - passive, or passive assisted, or active assisted movements of shoulder joint - scapulothoracic JCMT - active and passive movements of wrist joint, metacarpo-phalangeal joint, and interphalangeal joint FCMT - muscle release	^*^Conventional nursing care ^*^Medication Physical therapy	VAS	4.21 ± 1.15 → 1.56 ± 0.41	4.63 ± 0.121 → 5.03 ± 1.96	*p* < 0.05
	30 (12/18)	30 (16/14)	68.67 ± 8.17	65.79 ± 6.24				FMS	13.23 ± 11.31 → 43.12 ± 10.28	11.13 ± 11.39 → 17.54 ± 14.31	*p* < 0.05
								ER	90%	76.67%	*p* < 0.05
								AE	NR	NR	
Cheng (2015) (25)	EG	CG	EG	C	28 days 2 cycles (10 sessions/ cycle, 5 sessions/ week, 2-day rest/week)	^*^Medication ^*^Physical therapy ^*^Occupational therapy QD Control+ TCMT - pressing, kneading, rotating, grasping, finger twisting/holding, palm twisting/rubbing with two palms, and shaking - acupoints: LU1, LU5, LU6, PC7, PC6, PC1, HT1, HT3, HT5, HT7, HT8, HT9, LI4, LI10, LI11, LI14, LI15, TE5, TE13, TE14, EX-B2 (neck), BL11, BL12, BL13, BL14, BL15, BL16, BL17, BL41, BL42, BL43, BL44, BL45, BL46, SI3, SI8, SI11, SI15, GB21, and ashi points JCMT - scapulothoracic JCMT (passive or assisted active, or active movement)	^*^Medication ^*^Physical therapy ^*^Occupational therapy Suspension movement	VAS	7.19 ± 1.13 → 2.81 ± 0.71	6.86 ± 1.23 → 4.01 ± 0.82	*p* < 0.05
	30 (16/14)	30 (17/13)	55.31 ± 0.15	55.90 ± 11.30				FMS	33 ± 6.0 → 58 ± 7.0	33 ± 7.0 → 38 ± 5.0	*p* < 0.05
								BI	35.43 ± 7.13 → 59.55 ± 9.60	36.53 ± 8.72 → 53.64 ± 8.34	*p* < 0.05
								AE	NR	NR	
Lu (2015) (26)	EG	CG	EG	CG	29 days 2 cycles (14 sessions/ cycle, 1-day rest)	^*^Medication QD, 30 min Control+ TCMT - kneading, palm twisting/rubbing with two palms, and shaking - acupoints: GB21, LI15, SI9, 肩前, LI11, LI10, LI4, and SI3 JCMT - passive movements of upper limb	^*^Medication RH training	VAS	6.29 ± 2.27 → 3.37 ± 1.27	6.35 ± 2.24 → 4.91 ± 1.83	*p* < 0.05
	32 (18/14)	32 (16/16)	40–65	42–66				FMS	14.02 ± 4.82 → 35.41 ± 9.66	13.57 ± 5.09 → 27.46 ± 10.23	*p* < 0.05
								AE	NR	NR	
Sun (2015) (27)	EG	CG	30–60		NR	^*^Physical therapy BID Control+ TCMT - kneading (neck) FCMT - muscle release (including anterior scalene muscle and sternocleidomastoid muscle) Cervical exercise	^*^Physical therapy RH training	Edema (mL)	287.1 ± 24.5 → 245.8 ± 26	288.6 ± 19.8 → 268 ± 19.8	*p* < 0.05
	17	17						MPQ	34 ± 10.0 → 10 ± 7.0	34.5 ± 8.5 → 20 ± 11.0	*p* < 0.05
								AE	NR	NR	
Liu et al. (2011) (28)	EG	CG	EG	CG	3 cycles (15 sessions/cycle)	QD Control+ TCMT - kneading, pinching, palm twisting/rubbing with two palms, finger twisting/holding, and patting - acupoints: SI9, TE14, LI15, 肩 臑, LI11, LI10, SI5, TE4, LI5, and LI4 JCMT - passive and active movements of shoulder joint FCMT - ischemic compression - muscle resistance training	Routine RH training	VAS	60.63 ± 5.59 → 41.77 ± 5.29	59.97 ± 5.36 → 43.25 ± 5.67	*p* < 0.05
	30 (20/10)	30 (19/11)	58.15 ± 10.26	57.98 ± 10.91				FMS	14.90 ± 1.67 → 37.96 ± 3.62	14.77 ± 2.01 → 35.73 ± 3.98	*p* < 0.05
								Edema (score)	3.83 ± 2.15 → 1.32 ± 1.12	3.78 ± 2.08 → 2.45 ± 1.28	*p* < 0.05
								AE	NR	NR	

AE, Adverse Event; BI, Barthel Index; BID, Bis In Die; CG, Control Group; EG, Experimental Group; EOD, Each Other Day; ER, Effective rate; FCMT, Fascia Chuna Manual Therapy; FMS, Fugl-Meyer Score; MPQ, McGill Pain Quesitonnaire; NMS, Nailfole Microcirculation Score; NR, No Result; QD, Quaque Die; RH, Rehabilitation; SHSS, Shoulder Hand Syndrome Score; TCMT, Traditional Chuna Manual Therapy; JCMT, Joint-mobilizing Chuna Manual Therapy; VAS, Visual Analog Scale. ^*^indicates treatments provided as part of basic (conventional) care.

(4) Combination therapy (3 Studies)

Guo (29) included 64 patients who received conventional treatment and electroacupuncture for 11 days. The control group received rehabilitation training, whereas the experimental group received TCMT and JCMT. Significant between-group differences were observed in pain (*p* < 0.05).

Si et al. (30) treated 100 patients with rehabilitation training (proper limb positioning) for 34 days. The control group received traditional Chinese medicine fumigation, whereas the experimental group received additional TCMT and JCMT once daily for 25 min. The experimental group showed significantly greater reductions in pain and improvements in motor function compared with the control group (*p* < 0.05).

Zhang & Wang (31) included 51 patients aged 40–60 years who received conventional post-stroke treatment for 3 months. The control group underwent guided non-manipulative therapy, whereas the experimental group received additional TCMT, JCMT, and DCMT for 30 min every other day. ER was significantly higher in the experimental group than in the control group (*p* < 0.001).

Overall, all studies in this category reported significantly greater improvements in the experimental groups than in the control groups. When reported, the total effective rate was consistently higher in the experimental groups (Table 4).

**Table 4:** The analysis of 3 included studies that conducted manual therapy including Chuna as Combination therapy.

Author (year)	Number of SHS patients (Male/Female)		Age		Duration	Intervention	Control	Outcome measurements	Results		
									EG	CG	*P* value
Guo (2020) (29)	EG	CG	EG	CG	11 days 2 cycle (5 sessions/cycle, 1-day rest)	^*^Mediation ^*^Physical therapy ^*^Electroacupuncture TCMT - rolling, pressing, kneading, palm twisting/rubbing with two palms, and shaking - acupoints: Hand–Taiyang Small Intestine meridian, Hand–Shaoyang Triple Energizer meridian, SI9, SI10, TE13, LI15, 肩內陵, 举肩, TE4, ahi points, SI11 JCMT - passive movements of shoulder joint	^*^Medication ^*^Physical therapy ^*^Electroacupuncture RH training	FMS	29.30% (P25 = 28.00, P50 = 33.00, P75 = 36.75) → 32.78% (P25 = 46.00, P50 = 47.00, P75 = 49.00)	35.70% (P25 = 33.00, P50 = 35.00, P75 = 37.00) → 32.22% (P25 = 45.00, P50 = 47.00, P75 = 51.75	*p* > 0.05
	32 (14/18)	32 (11/21)	65.88 ± 2.061	63.44 ± 2.230				SHSS	31.39% (P25 = 9.00, P50 = 9.00, P75 = 10.00) → 25.19% (P25 = 3.00, P50 = 4.00, P75 = 6.11)	33.61% (P25 = 9.00, P50 = 10.00, P75 = 10.00) → 39.81% (P25 = 5.00, P50 = 6.00, P75 = 7.00)	*p* =0.001
								NRS	28.73% (P25 = 5.00, P50 = 7.00, P75 = 7.00) → 24.81% (P25 = 2.00, P50 = 3.00, P75 = 4.00)	36.27% (P25 = 6.25, P50 = 7.00, P75 = 8.00) → 40.19% (P25 = 3.00, P50 = 4.50, P75 = 6.00)	*p* = 0.001
								ER	90.63%	84.37%	*p* = 0.017
								AE	none	none	
Si et al. (2010) (30)	EG	CG	EG	CG	34 days 3 cycle (10 sessions/cycle, 2-day rest)	^*^Proper limb positioning training QD, 25 min TCMT - rolling, pressing, pushing, kneading, grasping, palm twisting/rubbing with two palms, and shaking - acupoints: GB21, LI15, 肩內陵, SI9, LI11, LI10, LI5, TE4, SI5, and LI4 JCMT - passive movements of upper limb	^*^Proper limb positioning training Traditional Chinese medicine fumigation	VAS	6.00 ± 4.03 → 1.67 ± 2.90	7.00 ± 4.05 → 3.40 ± 2.98	*p* < 0.05
	50 (30/20)	50 (28/22)	55 ± 7.12	56.72 ± 6.02				FMS	26.96 ± 7.25 → 48.31 ± 11.52	28.84 ± 8.32 → 43.06 ± 10.35	*p* < 0.05
								AE	NR	NR	
Zhang and Wang (2009) (31)	EG	CG	EG	CG	3 months	^*^Conventional post-stroke treatments (Not specified) EOD, 30 min TCMT - circular kneading, pinching, pressing, and kneading JCMT - passive movements of upper limb DCMT - shoulder	^*^Conventional post-stroke treatments (Not specified) Non-manipulation - guided active and passive movements of the affected limb	ER	90.0%	28.6%	*p* < 0.001
	30 (12/18)	21 (14/7)	40–60	40–60				AE	NR	NR	

AE, Adverse Event; BID, Bis In Die; CG, Control Group; DCMT, Dislocation Chuna Manual Therapy; EG, Experimental Group; EOD, Each Other Day; ER, Effective rate; FMS, Fugl-Meyer Score; NR, No Result; NRS, Numeric Rating Scale; QD, Quaque Die; SHSS, Shoulder Hand Syndrome Score; TCMT, Traditional Chuna Manual Therapy; VAS, Visual Analog Scale. ^*^indicates treatments provided as part of basic (conventional) care.

Across all intervention types, Tuina manual therapy consistently showed superior effects in reducing pain and improving upper limb motor function. Improvements in activities of daily living, range of motion, edema, and spasticity were also reported.

#### 3.2.6 Adverse events

Only two studies assessed adverse events, and none were reported. Yu (22) reported that no adverse events, including worsening of post-stroke shoulder–hand syndrome, skin allergy, skin damage, or ecchymosis, were observed during the study period. Guo (29) reported that no adverse events, such as skin allergy, infection at the massage sites, or local muscle strain, occurred.

## 4 Discussion

Stroke is a chronic condition characterized by the interruption of cerebral blood supply due to cerebrovascular disorders, leading to brain tissue damage and subsequent neurological dysfunctions (32). Approximately 66.0% of stroke patients experience severe post-stroke disabilities, manifesting as impairments in various domains such as language, cognition, sensation, perception, and motor functions, necessitating ongoing treatment and management (33). SHS is a sequela of stroke, presenting with symptoms including shoulder pain, hypersensitivity, dysesthesia, edema, and erythema. SHS not only delays the recovery of upper limb function but also deteriorates the quality of life for stroke patients (34, 35). If the condition progresses, it may lead to muscle atrophy and loss of motor function (36).

The etiology of secondary SHS remains unclear; however, it is hypothesized to result from shoulder and wrist damage due to improper upper limb movements during the early stages of stroke, impaired upper limb fluid circulation, and vascular dysfunction due to central nervous system damage (37). Particularly, the reduction in upper limb activity caused by SHS can perpetuate a vicious cycle of increased pain, underscoring the importance of timely intervention for symptom relief (38). Treatment for SHS includes pharmacological therapies, local anesthesia, and sympathetic nerve block, as well as non-pharmacological approaches such as supportive compression and rehabilitation exercises. However, there is no universally accepted single treatment modality for SHS (39). Additionally, it has been reported that healthcare costs increased by 2.17 times following an SHS diagnosis, with this increase persisting for at least 8 years (40), highlighting the need for standardized treatment guidelines for SHS.

In East Asian countries, manual therapy has developed under different systems, such as Tuina in China, Chuna in Korea, and Anma-massage-Shiatsu and Judo therapy in Japan. Chuna manual therapy has developed within the context of Korean medicine and shares historical and theoretical backgrounds with traditional Chinese Tuina. With the development of modern Chuna, it has evolved into a distinct system with its own classification framework and clinical applications. Furthermore, Chuna has developed as a practical discipline through the integration of traditional medical concepts, modern scientific knowledge, and various manual therapy techniques from different countries. Although all included studies employed Tuina, they met the inclusion criteria of this review, in which the applied manual techniques and underlying theoretical principles were consistent with those of Chuna. This approach reflects the conceptual and clinical overlap between Tuina and Chuna within East Asian medical systems, as described in prior studies (41–43).

In traditional Korean medicine literature, SHS is not directly mentioned. However, it can be considered within the category of “Bi Syndrome” resulting from stroke. Bi Syndrome is characterized by Qi and Blood Stagnation, leading to pain, swelling, and motor dysfunction, with severe cases potentially resulting in joint deformity (44). Recent research by Huang et al. (45) has suggested through meta-analysis that non-pharmacological treatments for SHS may demonstrate significant efficacy compared to conventional treatments. In South Korea, research trends regarding acupuncture for SHS have been reported (46), and international research trends on acupuncture for SHS have also been documented (47). However, studies evaluating the efficacy of manual therapy including Chuna have not been conducted, thus prompting this study. Chuna therapy involves the application of manual techniques by a practitioner to specific areas of the body based on traditional Korean medical principles, such as activating Qi and Blood circulation, restoring muscle and joint alignment, enhancing joint mobility, regulating internal organs, and strengthening the body's resistance while expelling pathogenic factors. This therapy aims to regulate physiological and pathological conditions and has the potential to improve joint imbalance and functional recovery in musculoskeletal disorders, suggesting its potential as an effective treatment for SHS (9).

A total of 13 studies were selected, each implementing various Tuina manual therapy techniques or procedural sequences but all applied manual therapy incorporating Chuna manual therapy. The treatment duration ranged from 11 days to 4 months. Regarding treatment frequency, manual therapy was performed twice, once daily and every other day, while 1 study did not report the frequency. The duration of each treatment session was ranged from 15 to 30 min.

Although the Chuna techniques used varied across studies, TCMT was applied in most of them. These techniques can be further categorized according to their specific therapeutic actions. Pressing (19–23, 25, 29–31), grasping (25, 30), pushing (22, 30), rolling (22, 23, 29, 30), and kneading (23, 25–31) were classified as FCMT, whereas shaking (25, 26, 29, 30), rotating (23, 25), and finger twisting/holding (25, 28) were categorized as JCMT. Circular kneading (19, 20, 23, 31) was considered to correspond to musculotendinous releasing manual therapy. In contrast, palm twisting/rubbing with two palms (22, 23, 25, 26, 28–30), slipping/wiping (22), pinching (28, 31), and patting (28) are not regarded as modern Chuna techniques (9). Overall, FCMT was the most frequently applied Chuna technique, followed by JCMT and musculotendinous releasing manual therapy. These findings indicate that FCMT was the most commonly used modern Chuna technique for SHS. Unlike osteopathic Chuna, FCMT primarily targets soft tissues, including muscles, tendons, ligaments, and fascia. It encompasses various therapeutic approaches such as muscle and fascial compression, myofascial relaxation, muscle strengthening and relaxation, ligamentous joint relaxation, fascial diaphragm techniques, and muscle stretching (9). Previous studies have suggested the suitability of FCMT for patients with stroke (48), and the frequent use of FCMT in the included studies may reflect its clinical applicability in managing SHS, which is characterized by pain, edema, and soft tissue dysfunction.

In addition to FCMT, other modern Chuna techniques such as JCMT and DCMT may have distinct clinical roles in the management of SHS. JCMT is applied to restore joint malalignment and range-of-motion abnormalities that arise from impaired circulation of qi and blood, which leads to functional restriction of the affected joint (9). Considering that the traditional Korean medicine pathophysiology of SHS is also associated with disturbances in qi and blood circulation, JCMT may be clinically applicable for improving joint mobility and functional recovery in patients with SHS. DCMT was reported in one study and may be considered when shoulder dislocation occurs during the progression of SHS, as it aims to achieve joint reduction and restore structural alignment. Taken together, these findings suggest that different Chuna techniques may be selectively applied according to the clinical presentation and stage of SHS.

In several included studies, manual therapy was applied to specific acupoints. A total of 47 acupoints were identified, with 96 applications in total. When analyzed by anatomical region, the shoulder was the most frequently treated area (GB21, HT1, LI14, LI15, LU1, PC1, SI9, SI10, SI11, SI15, TE13, TE14, Jianqian, Jianneiling, and Jianzhen; 37 applications), followed by the elbow (HT3, LI10, LI11, LU5, LU6, and SI8; 18 applications), the wrist (HT5, HT7, LI5, PC6, PC7, SI5, TE4, and TE5; 14 applications), the hand (HT8, HT9, LI4, SI3, and EX-UE9; 13 applications), and the spine (EX-B2 [cervical], BL11–BL17, and BL41–BL46; 14 applications). All studies included acupoints located in the shoulder region, indicating that manual therapy primarily focused on the shoulder joint. This distribution may reflect the pathophysiological characteristics of post-stroke SHS. Previous research (49) has reported that increased instability of the shoulder and wrist joints due to reduced muscle tone in stroke patients can cause damage to surrounding soft tissues and potentially lead to SHS. In this context, FCMT targeting the upper limb, particularly the shoulder region, may be clinically relevant.

Recent advances in neurorehabilitation have emphasized the importance of multimodal rehabilitation approaches and the role of neuroplasticity in functional recovery. In particular, functional recovery is increasingly understood as a result of interactions between various therapeutic inputs and task-specific training, rather than a single intervention (50). Within this framework, Tuina (or Chuna) manual therapy may be interpreted as a complementary component that can be integrated with conventional rehabilitation strategies. This perspective may also help explain the variability in treatment outcomes observed across the included studies.

Although most of the included studies reported significant improvements in clinical outcomes, several methodological limitations should be considered when interpreting these findings. First, while randomization using a random number table was reported in a number of studies, information on allocation concealment and blinding was generally lacking. Given the nature of manual therapy, participant and practitioner blinding is inherently difficult. However, the absence of assessor blinding may have increased the risk of performance and detection bias.

Second, the outcome measures used across the studies were heterogeneous, including pain intensity, upper limb motor function, activities of daily living, edema, range of motion, spasticity, and total effective rate. In particular, the total effective rate, which was frequently reported, is neither a standardized nor an internationally validated outcome measure, and its definitions and evaluation criteria varied across studies. This lack of standardization limits comparability and hinders accurate estimation of treatment effects.

Furthermore, the reliance on narrative synthesis reflects substantial clinical and methodological heterogeneity among the included studies. Variations in intervention protocols, co-interventions, and treatment durations precluded quantitative meta-analysis. As a result, the findings should be interpreted with caution. Collectively, these issues may introduce potential bias, thereby limiting the internal validity of the evidence and reducing the generalizability of the conclusions.

Third, the combination of co-interventions differed considerably among studies. Tuina manual therapy was applied as monotherapy, as an add-on to traditional Chinese medicine, as an adjunct to conventional rehabilitation, or as part of combination therapy. Such clinical heterogeneity makes it difficult to determine the independent effect of Tuina manual therapy.

In addition, all included studies were conducted in China, which may limit the generalizability of the findings to other healthcare systems and clinical settings. All interventions were described as Tuina-based manual therapy and were further analyzed and interpreted within the framework of Chuna manual therapy; however, this interpretative approach may introduce potential heterogeneity and should be considered when interpreting the findings.

In terms of safety, only two studies assessed adverse events, and no treatment-related adverse events were reported. However, the limited assessment and reporting of safety outcomes make it difficult to draw definitive conclusions regarding the safety profile of Tuina manual therapy. The absence of reported adverse events should therefore be interpreted with caution, as underreporting or inadequate monitoring cannot be ruled out.

Taken together, these findings suggest that although Tuina manual therapy may have beneficial effects for post-stroke shoulder–hand syndrome, the overall certainty of the evidence is limited by methodological shortcomings and insufficient safety reporting. Future well-designed randomized controlled trials with rigorous allocation concealment, assessor blinding, standardized outcome measures, and systematic monitoring of adverse events conducted in diverse clinical settings are required to establish both the effectiveness and safety of this intervention.

## 5 Conclusion

This study aimed to evaluate the clinical efficacy and safety of manual therapy including Tuina therapy for post-stroke SHS. Through the review of 13 randomized controlled trials (RCTs), manual therapy including Tuina (or Chuna) appeared to be beneficial for reducing pain and improving upper limb function in patients with SHS. Among the various techniques, FCMT was most frequently applied, followed by JCMT and DCMT, suggesting their potential clinical relevance. However, the overall certainty of the evidence remains limited due to substantial clinical and methodological heterogeneity, variations in intervention strategies, and the exclusive inclusion of studies conducted in China, which may restrict the generalizability of the findings. Therefore, the current evidence should be interpreted with caution. Further high-quality studies are required to establish standardized treatment guidelines.
